# Supplementary material for: Two telomere-to-telomere gapless genomes reveal insights into Capsicum evolution and capsaicinoid biosynthesis
Source: Nat Commun. 2024 May 20;15:4295. doi: 10.1038/s41467-024-48643-0 (PMC11106260; doi:10.1038/s41467-024-48643-0)
Supplement: Supplementary file 1 — Supplementary Information [file 41467_2024_48643_MOESM1_ESM.pdf]

Two telomere-to-telomere gapless genomes reveal insights into *Capsicum* evolution and capsaicinoid biosynthesis

Chen *et al*.

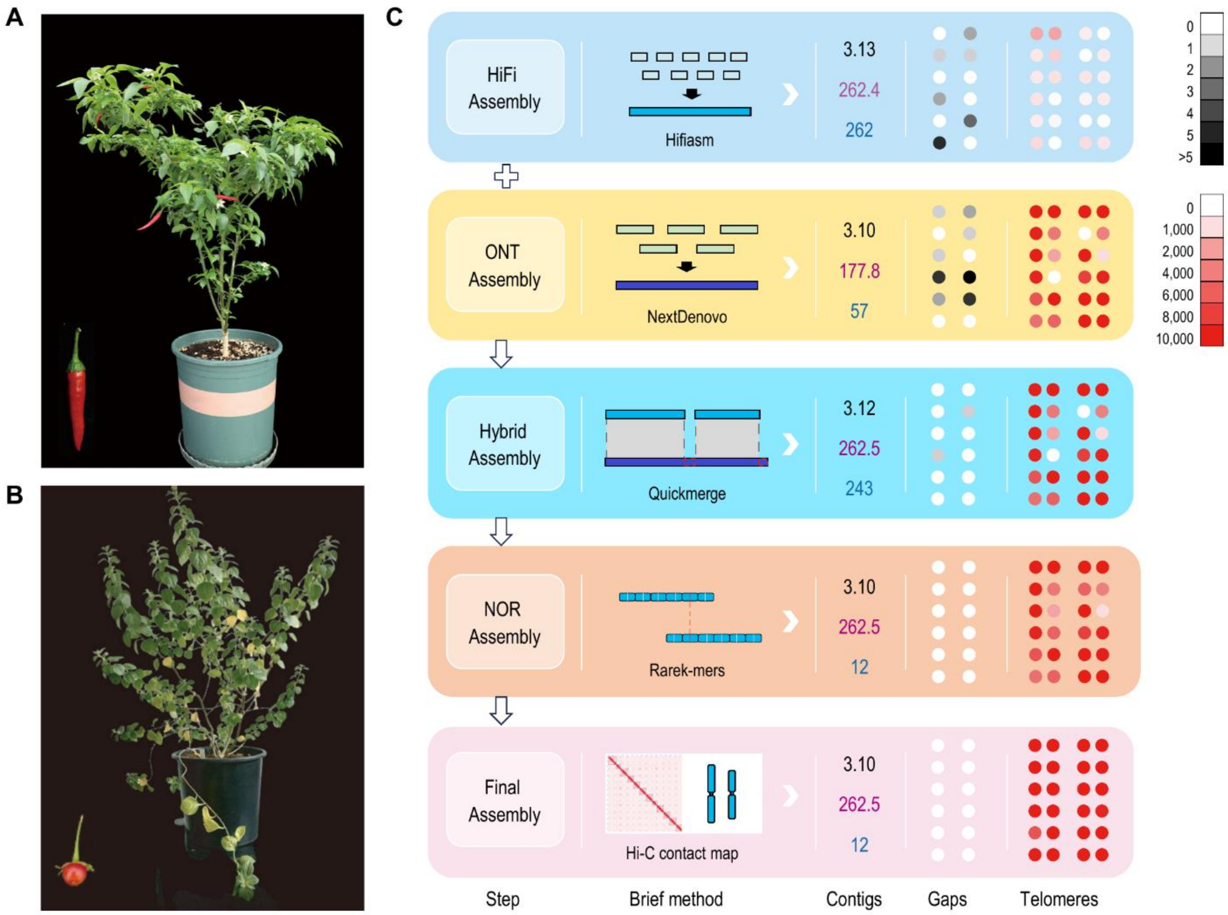

Supplementary Fig. 1. Pepper genome assembly pipeline for C. annuum and C. rhomboideum. (A-B) An image of the chili pepper *C. annuum* (A) and its wild nonpungent relative *C. rhomboideum* (B)**. (C)** Computational pipeline for T2T gap-free genome assembly of *C. annuum*. The number above "Contigs" means assembly size, contig N50 and contig number; the black circles above "Gaps" means gap number in 12 chromosomes; and the red circles above "Telomeres" means copy numbers of seven-base telomeric repeat in 24 telomere regions.

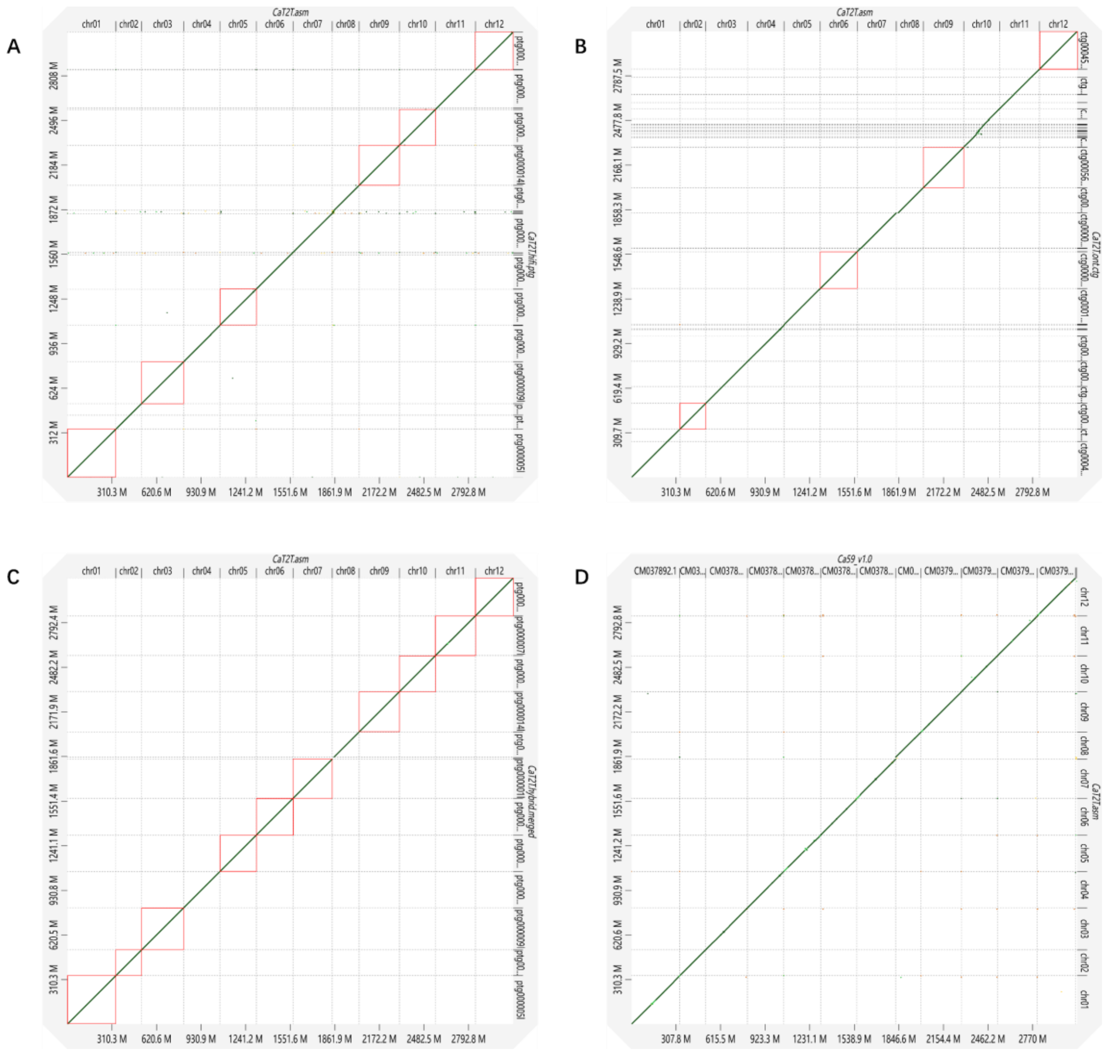

Supplementary Fig. 2. Alignment of *C. annuum* genome assemblies. (**A**) Syntenic dot plot between the HiFi assembly and the final CaT2T assembly. The red box means T2T contigs assembled by Hifiasm using only HiFi data. (**B**) Syntenic dot plot between the ONT assembly and the final CaT2T assembly. The red box means T2T contigs assembled by NextDenovo using only ONT data. (**C**) Syntenic dot plot between the hybrid assembly and the final CaT2T assembly. The red box means T2T contigs. (**D**) Syntenic dot plot between the final CaT2T assembly and the reported Ca59 reference.

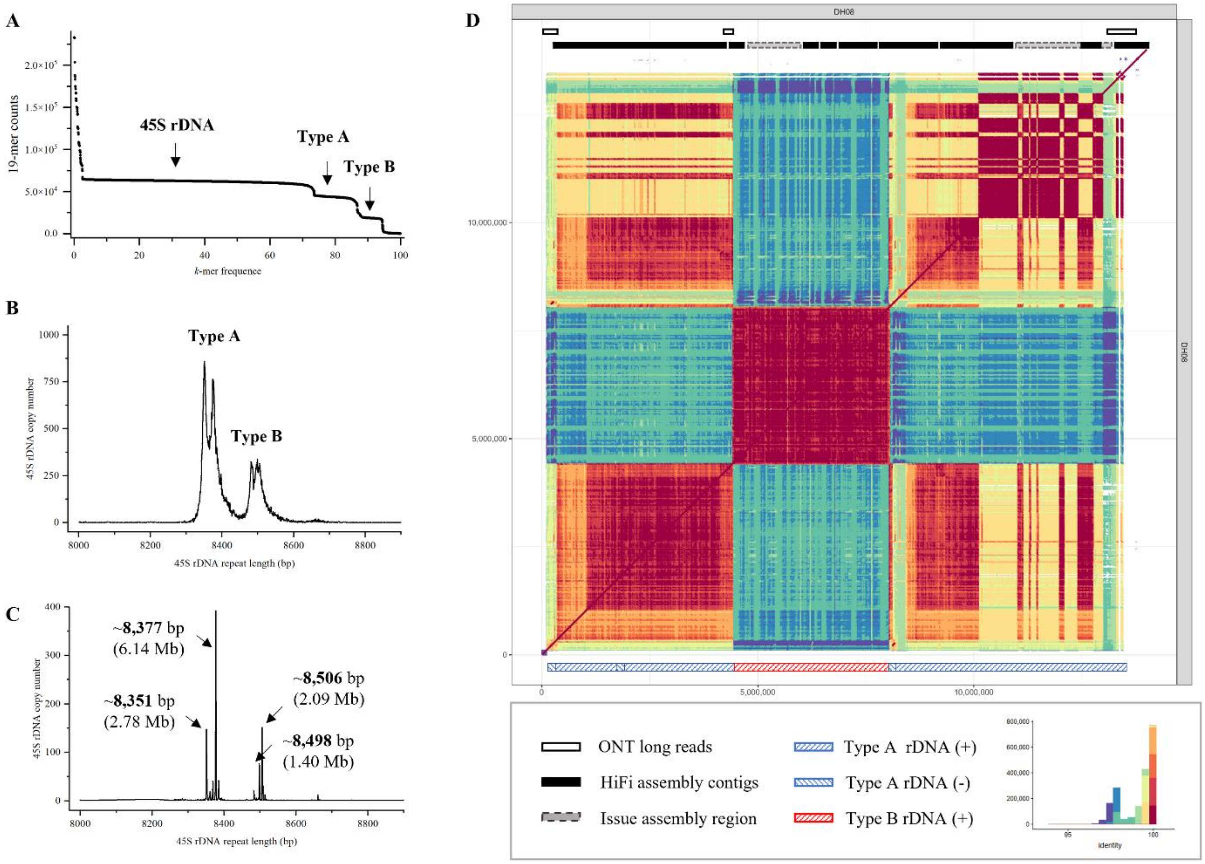

Supplementary Fig. 3. Estimating rDNA copy number and overview of rDNA arrays in *C. annuum*. (**A**) The 45S rDNA-containing reads extracted from 42× HiFi (>20 kb) data were used to conduct 19-mer analysis, and the rDNA arrays were estimated to be 1,428 copies (60,000 counts / 42× depth); (**B**) The 45S rDNA-containing reads were partitioned into units, where each unit represents a single repeat copy. These units varied on the repeat length and could be divided into two major types; (**C**) Different types of 45S rDNA repeat unit were counted and summed in the final T2T assembly; (**D**) Pairwise sequence identity across the NOR region based on non-overlapping 5 kb window size. The above rectangle meant the assembly was based on ONT long reads and HiFi contigs. Two issue regions corresponding to multiple Type A and Type B contigs were assembled using Hi-C interaction matrix. The below rectangle meant the (+) or (-) direction of each rDNA repeat unit.

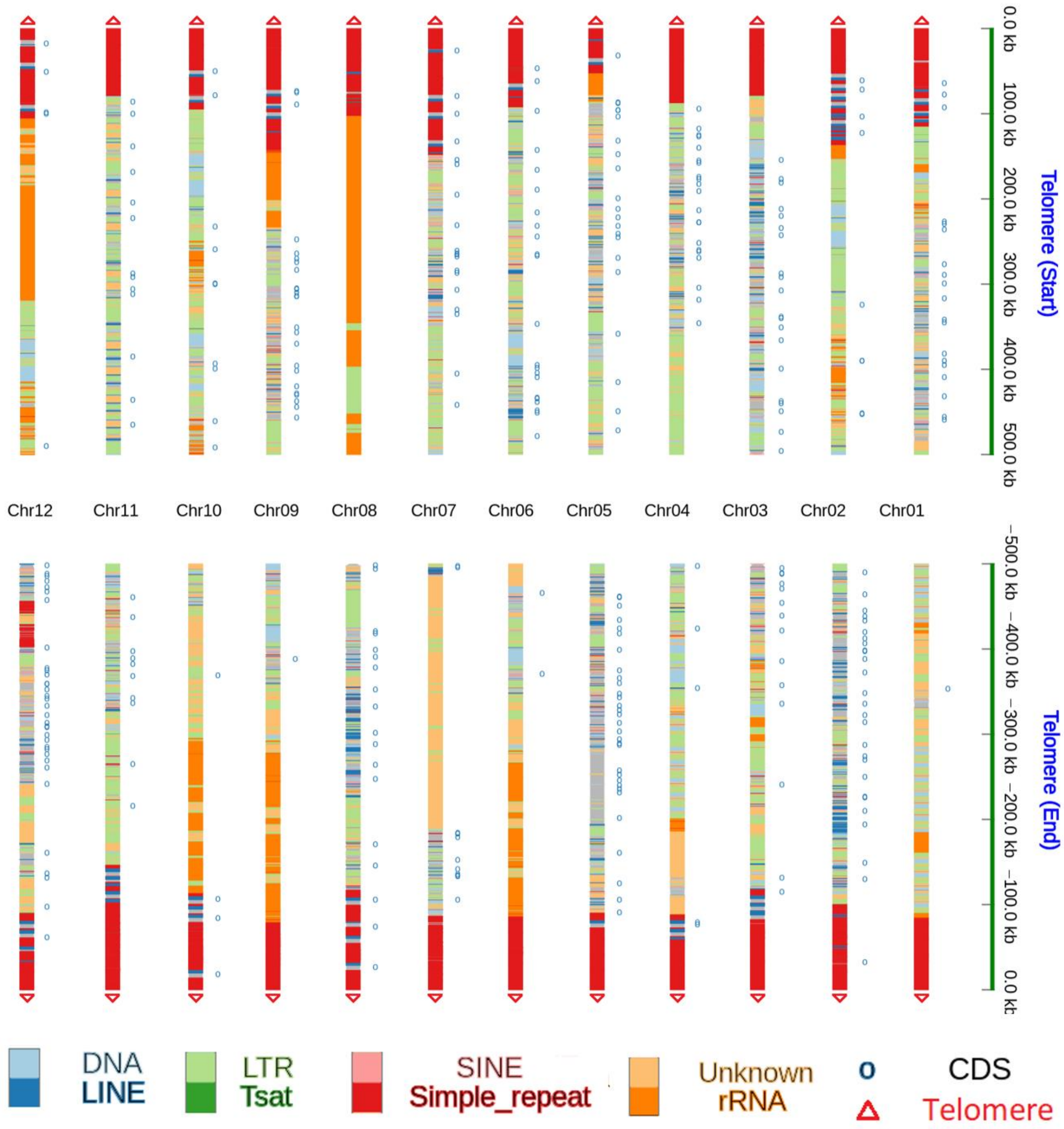

Supplementary Fig. 4. The composition and distribution of repetitive sequences and protein-coding genes in *C. annuum* telomeric 500 kb regions. DNA: DNA transposons. LINE: long interspersed nuclear elements. LTR: long terminal repeats. SINE: short interspersed nuclear elements.

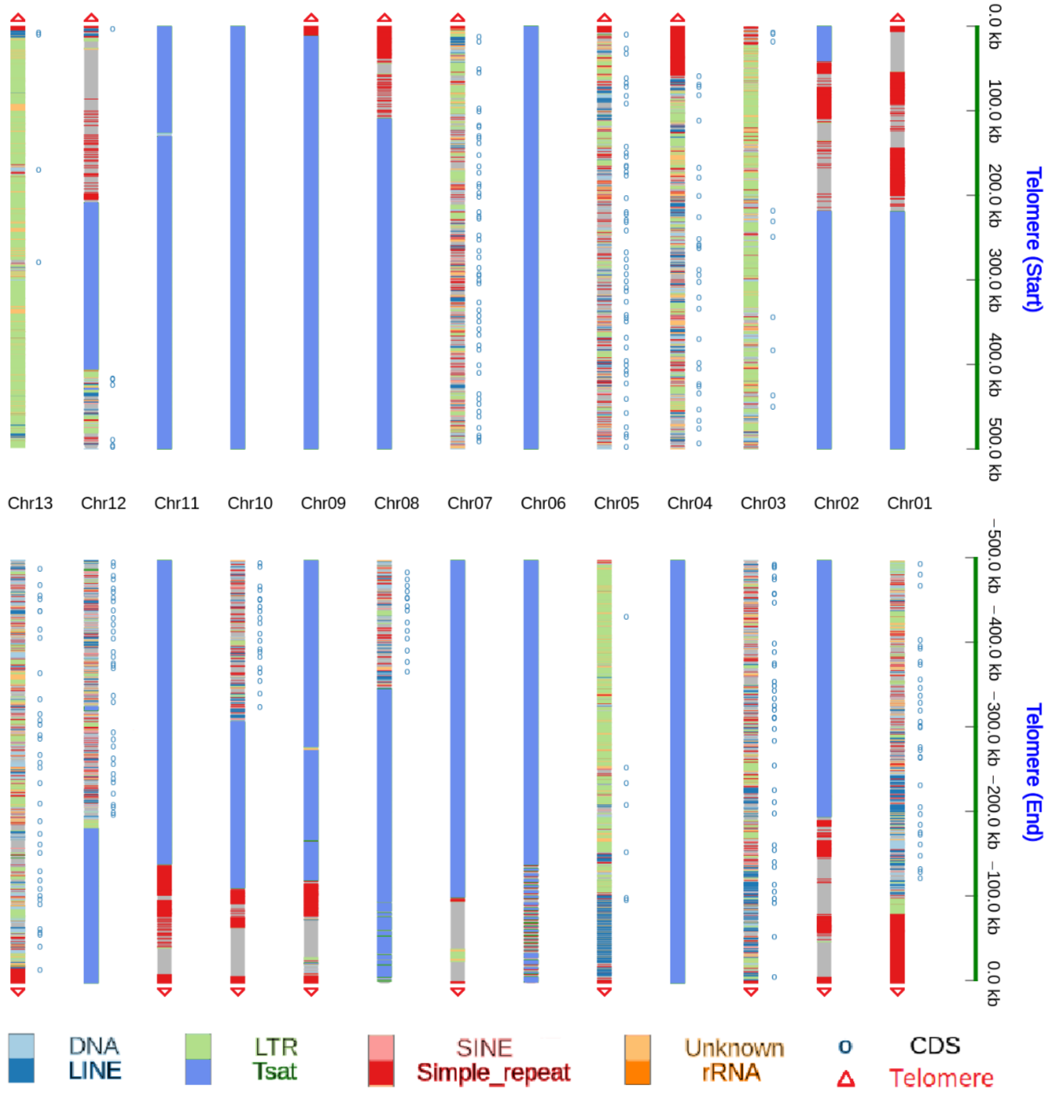

Supplementary Fig. 5. The composition and distribution of repetitive sequences and protein-coding genes in *C. rhomboideum* telomeric 500 kb regions. DNA: DNA transposons. LINE: long interspersed nuclear elements. LTR: long terminal repeats. Tsat: 183-bp telomeric-specific satellites. SINE: short interspersed nuclear elements.

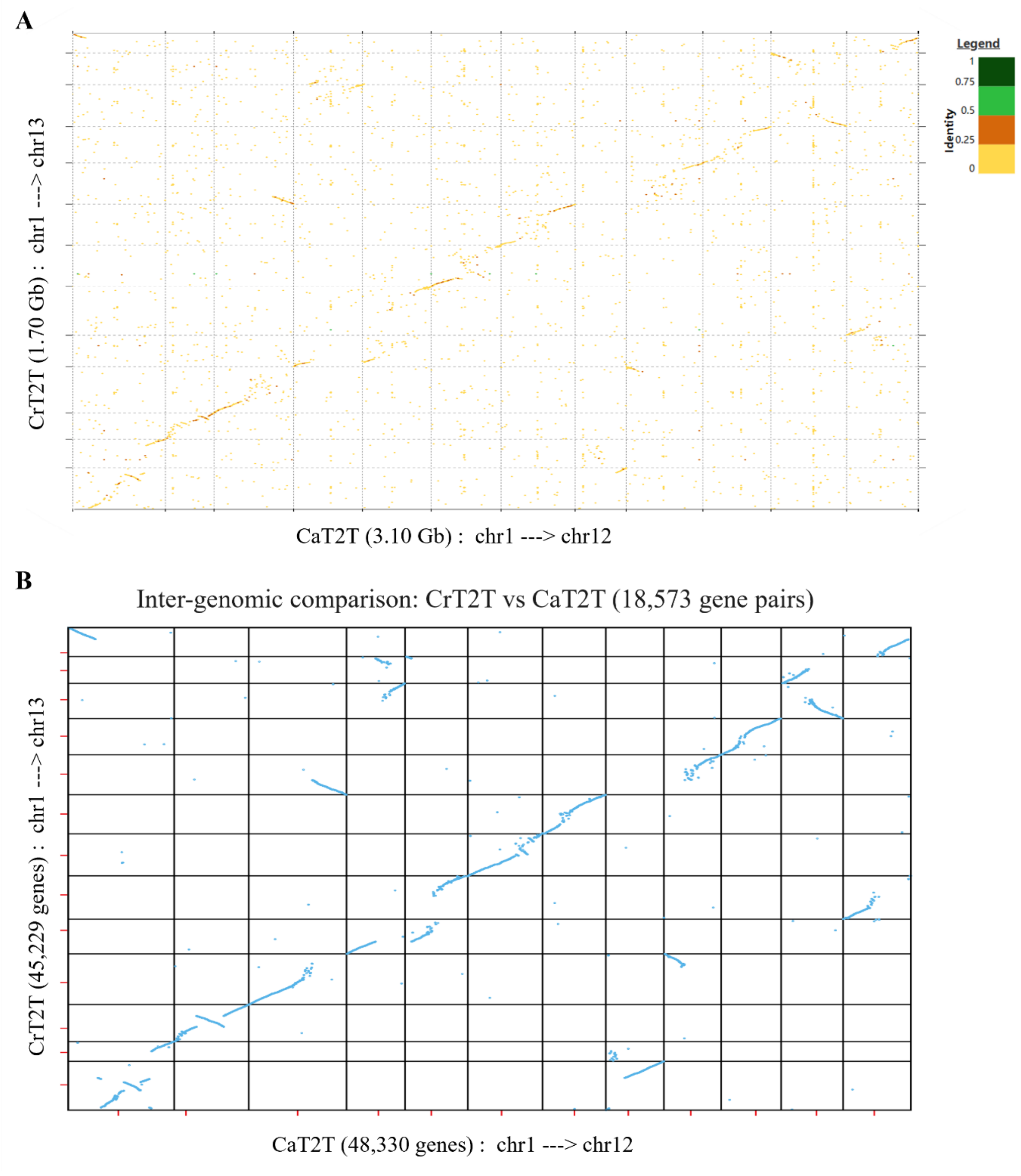

Supplementary Fig. 6. Whole genome alignments of C. annuum and C. rhomboideum. (**A**) Genomic alignment dot plot between the CaT2T and CrT2T assemblies generated using Minimap2 and D-GENIES. (**B**) Dot plots of interspecies syntenic blocks of CaT2T and CrT2T. Each dot indicates a syntenic gene pair detected by MCScanX.

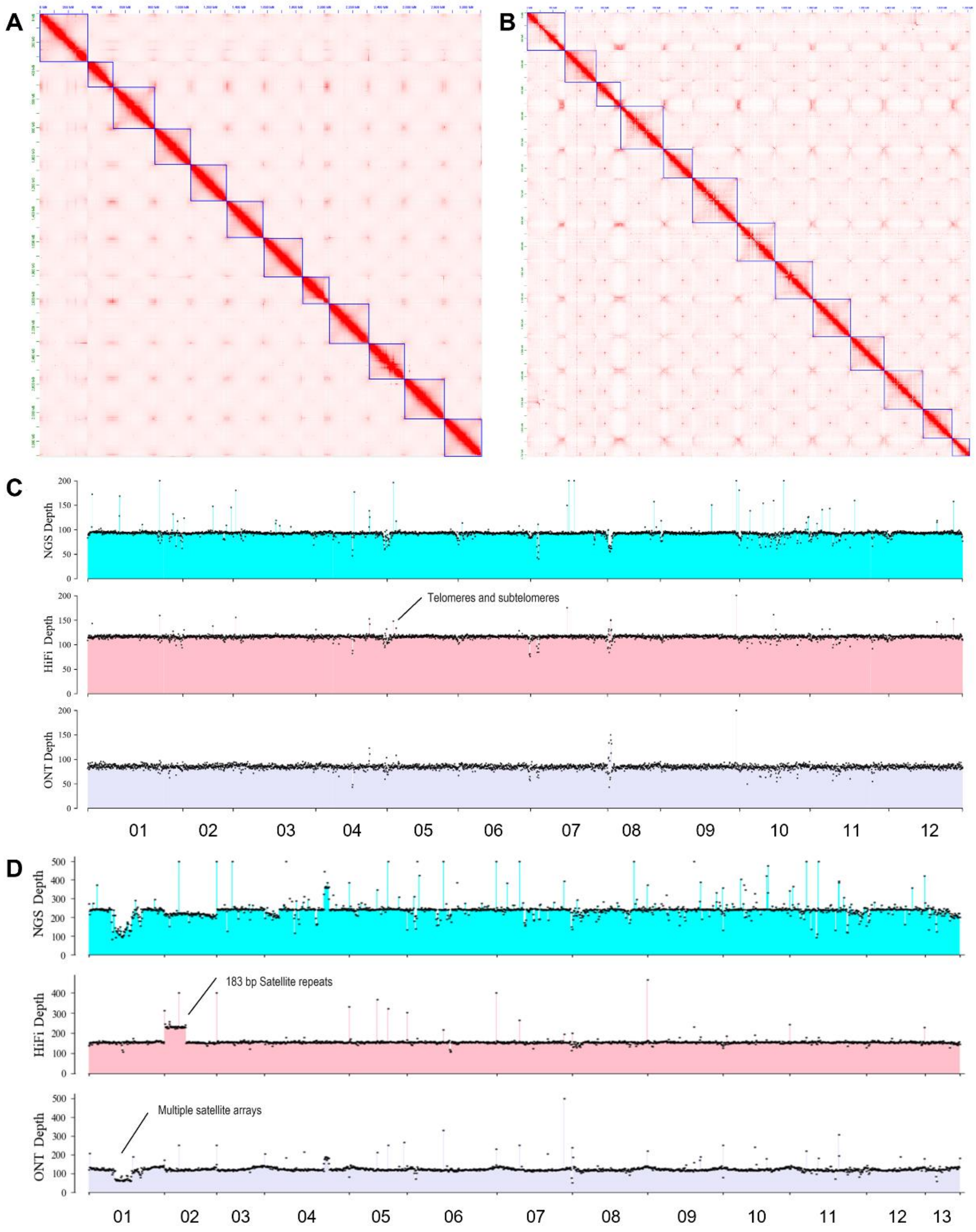

Supplementary Fig. 7. Overview of the Hi-C count matrix and sequencing coverage depth for the CaT2T and CrT2T genome. (**A-B**) Hi-C interaction matrix of CaT2T (**A**) and CrT2T (**B**). Chromosomes were anchored in order from top to bottom: Chr01 to Chr12 (CaT2T) and Chr01 to Chr13 (CrT2T). (**C-D**) NGS, HiFi and ONT coverage depth in CaT2T (**C**) and CrT2T (**D**). The abnormal low or high coverage depth was due to repetitive sequences as telomeres/subtelomeres and multiple long arrays of satellite repeats.

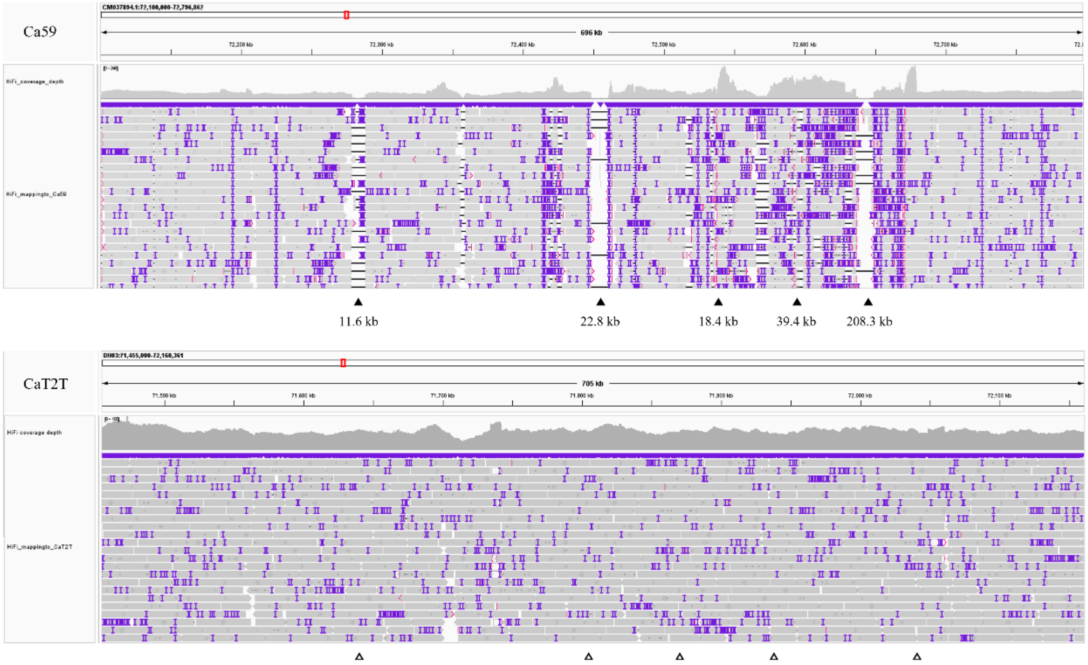

Supplementary Fig. 8. An example of closed gaps in CaT2T compared to Ca59. HiFi reads were mapped to Ca59 and CaT2T genome using Minimap2 (-x asm5) and the coverage depth in Chr03: 71.45-72.16 Mb was manually checked in IGV.

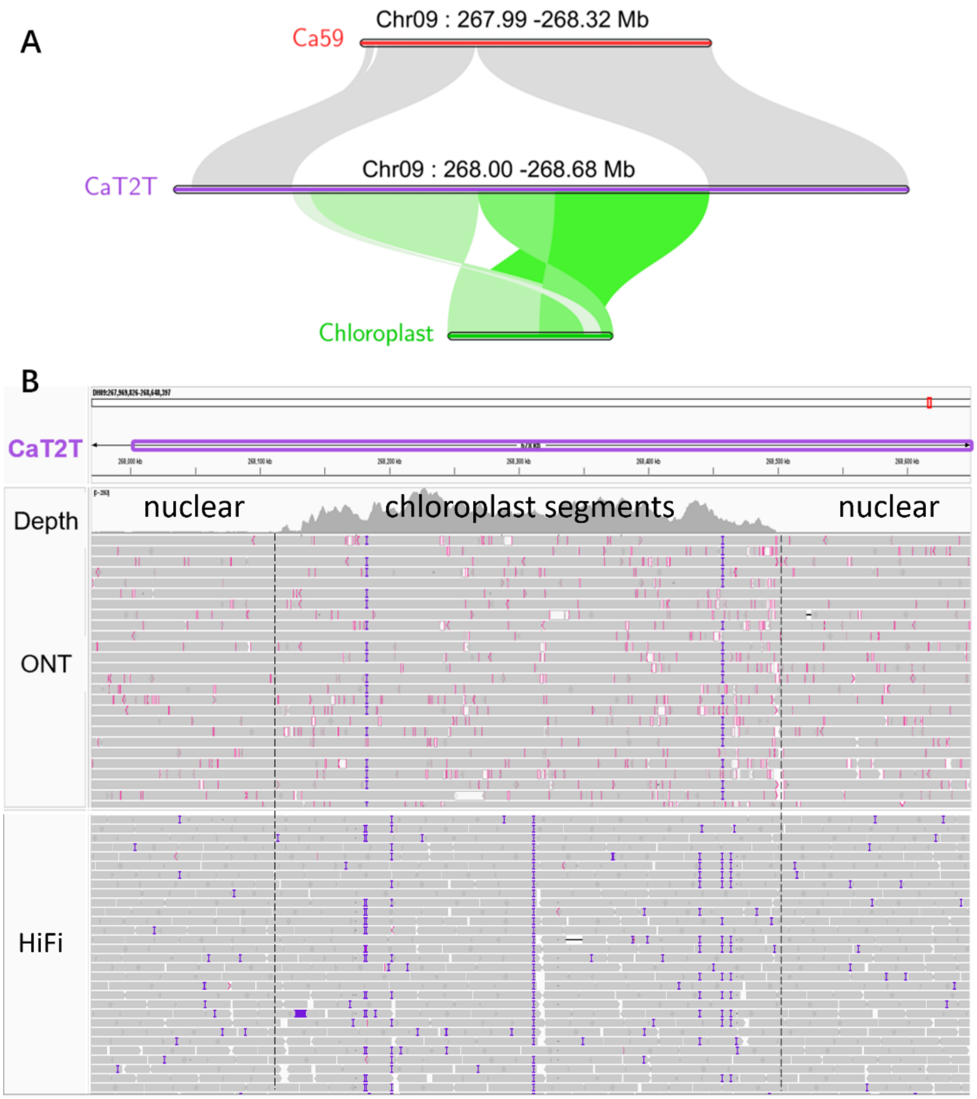

Supplementary Fig. 9. Nuclear chloroplast and mitochondria DNA insertion in CaT2T assembly. (**A**) An example of chloroplast DNA insertion in CaT2T nuclear Chr09: 268.10-268.50 Mb. Synteny comparison between Ca59, CaT2T and chloroplast (NC_018552.1) of pepper. (**B**) The coverage of ultra-long ONT and HiFi reads across the chloroplast DNA insertion site at Chr09: 268.10-268.50 Mb.

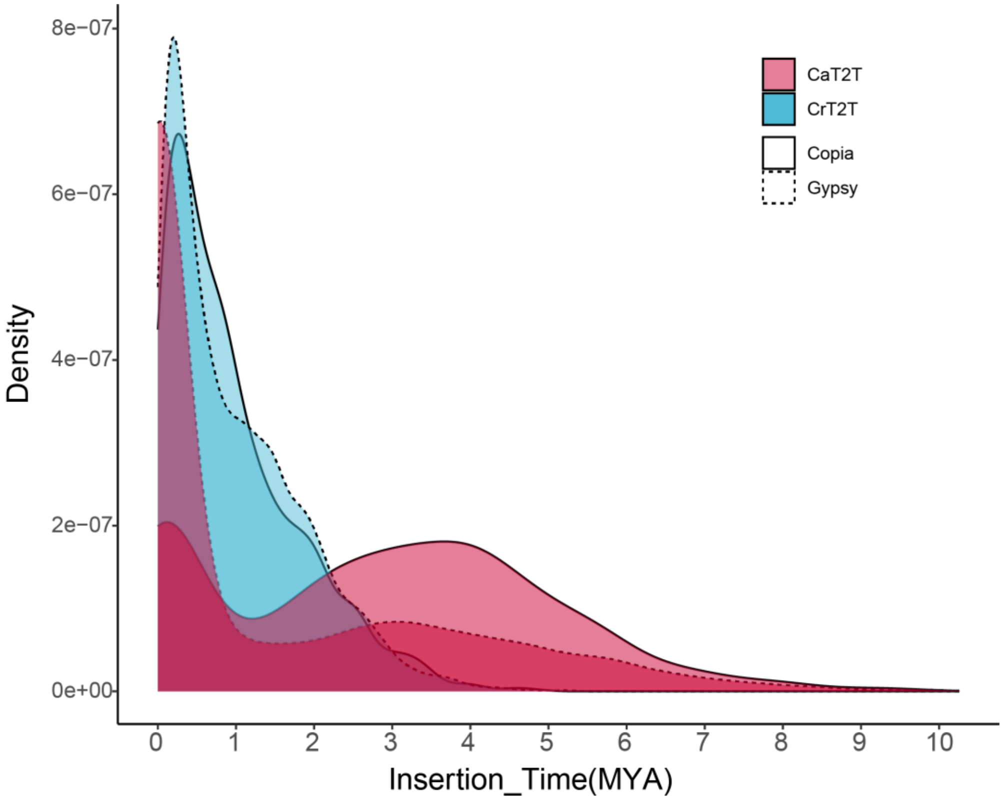

Supplementary Fig. 10. Distribution of insertion times of intact *Gypsy* and *Copia* retrotransposons in CaT2T and CrT2T assemblies.

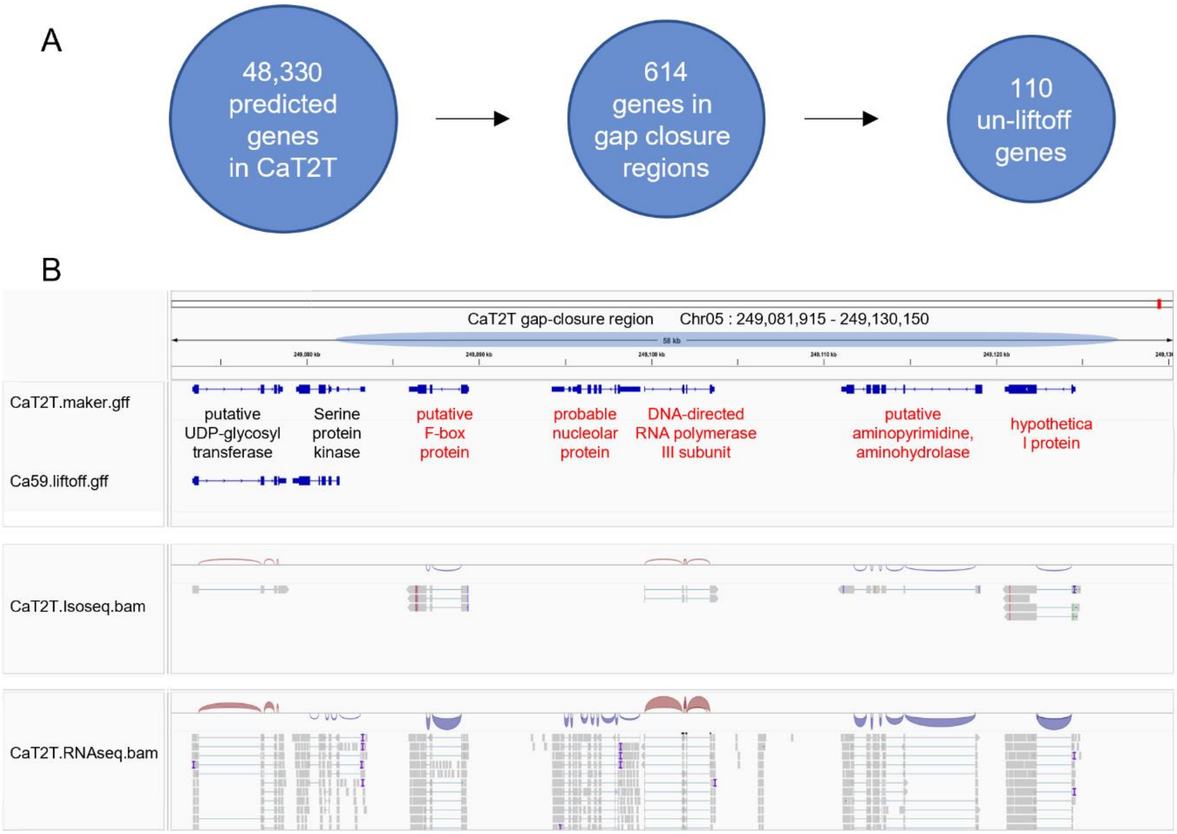

Supplementary Fig. 11. An example of novel annotated gene models in gap-closure regions in CaT2T. (**A**) Among the 614 genes located in gap-closure regions, 110 were unmapped from a CaT2T reference genome to Ca59 using Liftoff with parameters of '-copies -flank 0.1 -sc 0.9'. (**B**) An example of the highly expressed novel genes in gap-closure regions of Chr05: 249.08 Mb -249.13 Mb.

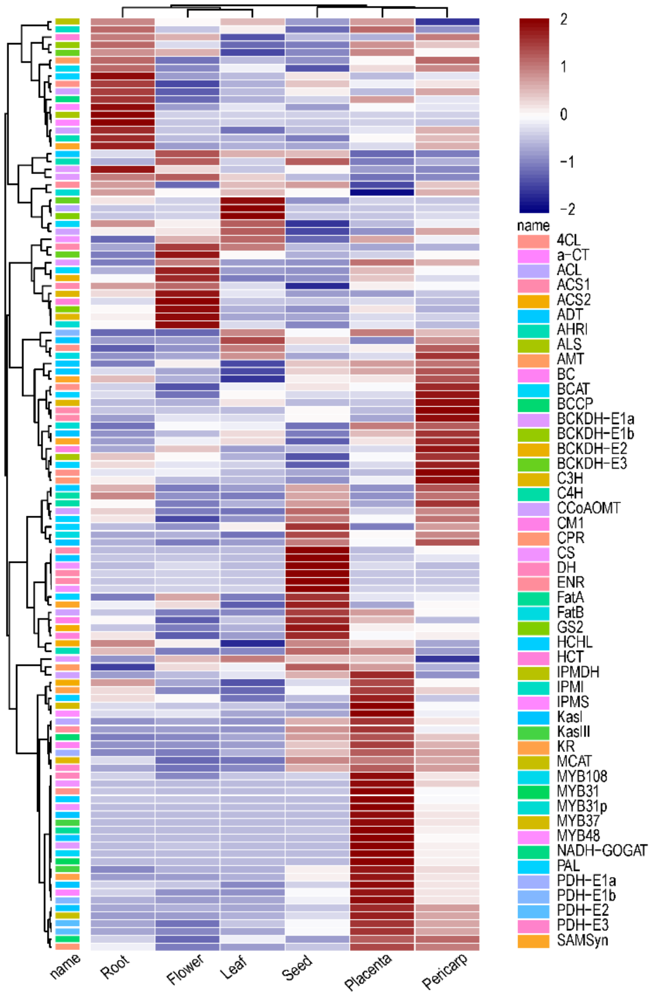

Supplementary Fig. 12. Expression heatmap of putative CBGs in different tissues of *C. annuum.*

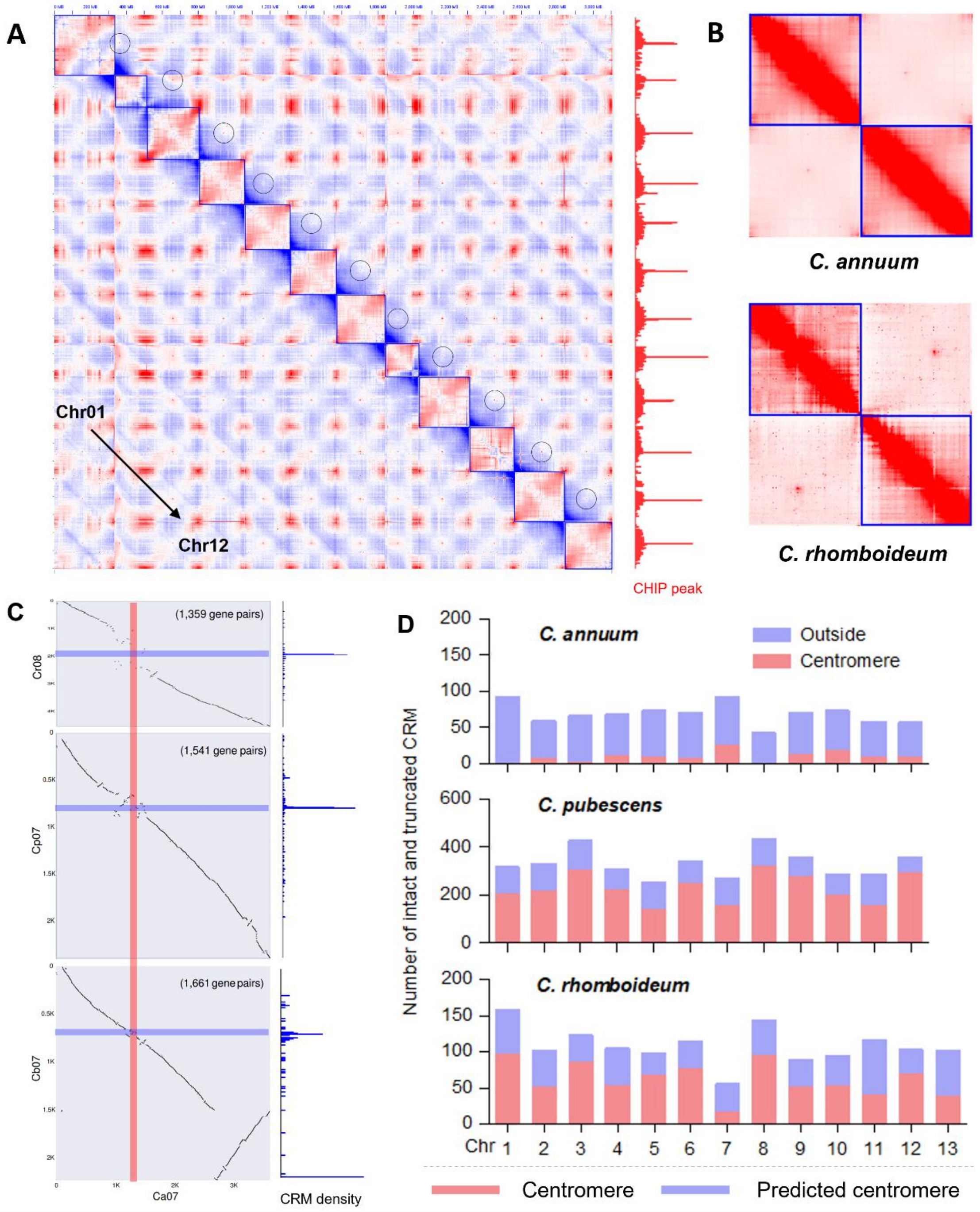

Supplementary Fig. 13. Prediction of centromeres in *Capsicum* assemblies without CHIP-seq data. (**A**) The inter-chromosome interactions were positively correlated with the ChIP peak in *C. annuum*. (**B**) The *C. rhomboideum* showed higher inter-chromosome interactions than *C. annuum*. (**C**) The predicted centromeres in *C. baccatum*, *C. pubescens* and *C. rhomboideum were* positively correlated with the CRM density. (**D**) The enrichment of CRM in centromeres, especially in *C. pubescens* and *C. rhomboideum.*

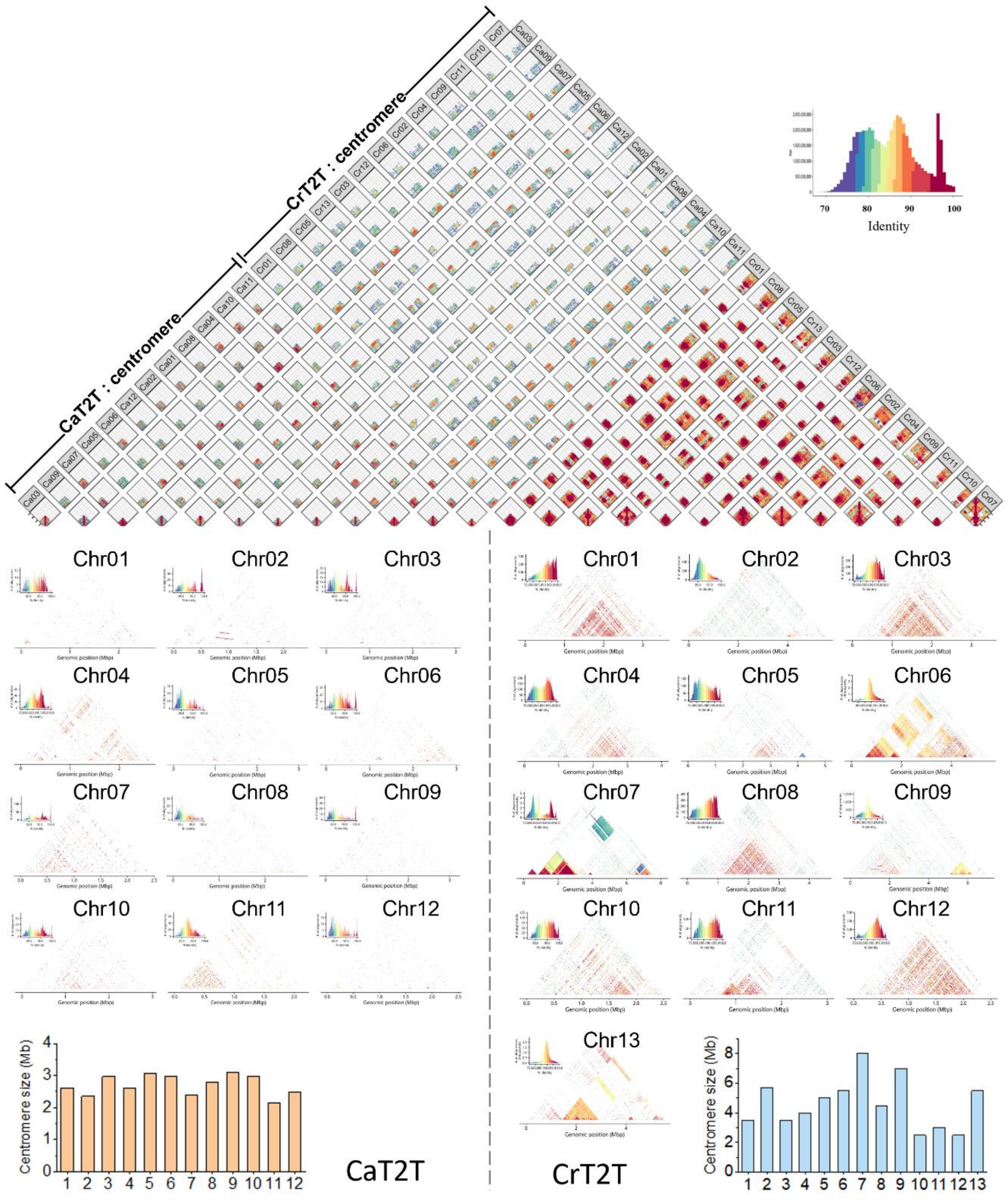

Supplementary Fig. 14. **Pairwise sequence identity heatmap in centromeres of two *Capsicum* T2T assemblies.** The non-overlapping 5 kb bins were aligned with each other using minimap2, and only the best local alignment between each pair was retained. Sequence identity was calculated from the highest scoring alignment between each pair, and was shown using StainedGlass. The centromere length in *C. annuum* were identified by ChIP-seq, while the centromeres in *C. rhomboideum* were predicted by CRM retrotransposon distribution.

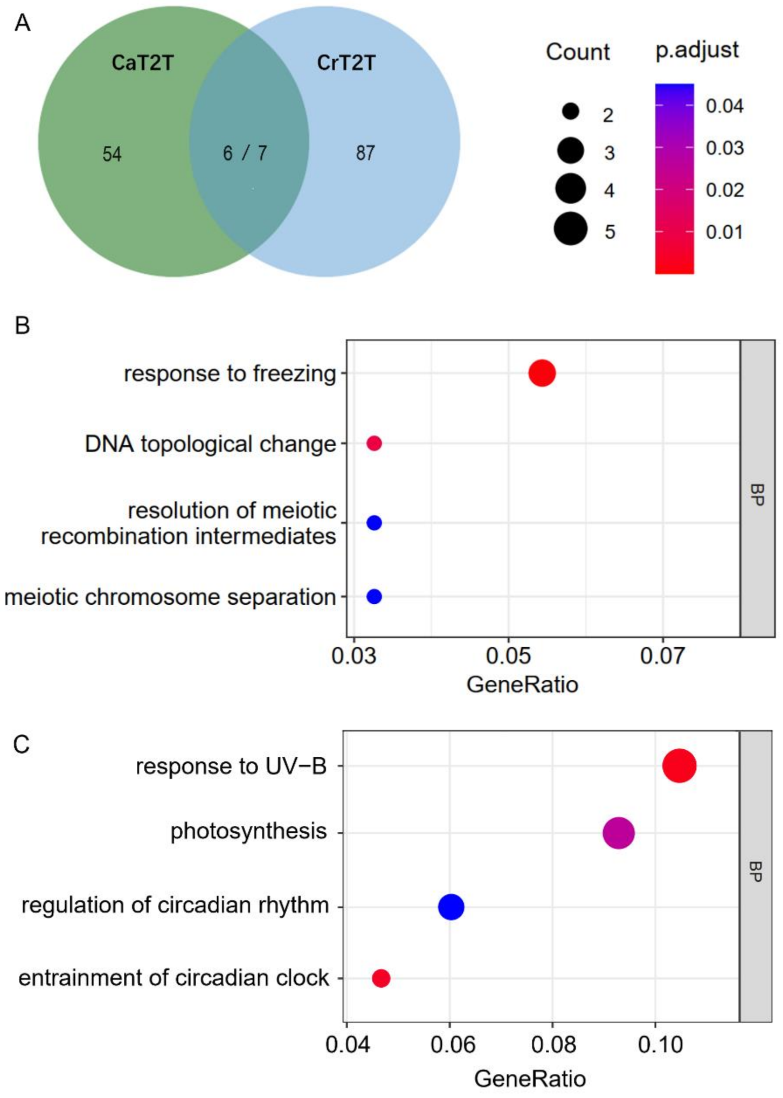

Supplementary Fig. 15. GO enrichment analysis of annotated genes in centromere regions. (**A**) Venn diagram of common proteins in *C. annuum* and *C. rhomboideum* centromere regions. (**B**) GO enrichment analysis of 252 genes in *C. annuum* centromere regions. (**C**) GO enrichment analysis of 386 genes in *C. rhomboideum* centromere regions.

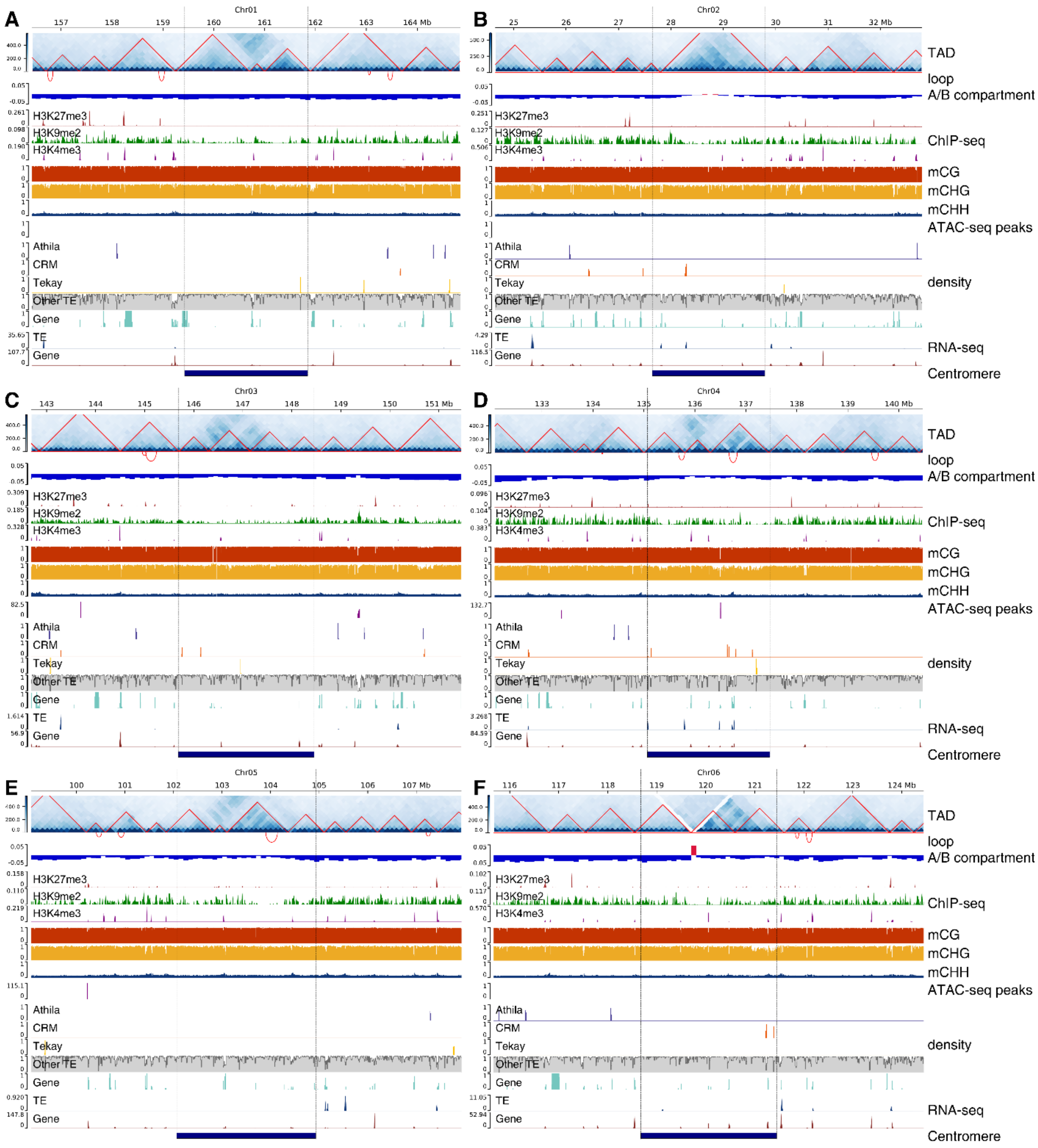

Supplementary Fig. 16. Characteristics of the centromeres on Chr01-Chr06 of *C. annuum*. (**A**-**F**) Characteristics of different centromeres in *C. annuum* assembly. The distributions of TAD and chromatin loops, A/B compartment, ChIP-seq signals (H3K27me3, red; H3K9me2, green; H3K4me3, purple), methylation levels (CG, red; CHG, green; CHH, purple), ATAC-seq signal, TE elements (Athila, bule; CRM, yellow; Tekay, red; others, grey), gene density (green), TE transcript abundances (blue) and gene transcript abundances (red) were plotted from the top to bottom successively. The centromere region was marked by the bottom blue rectangle.

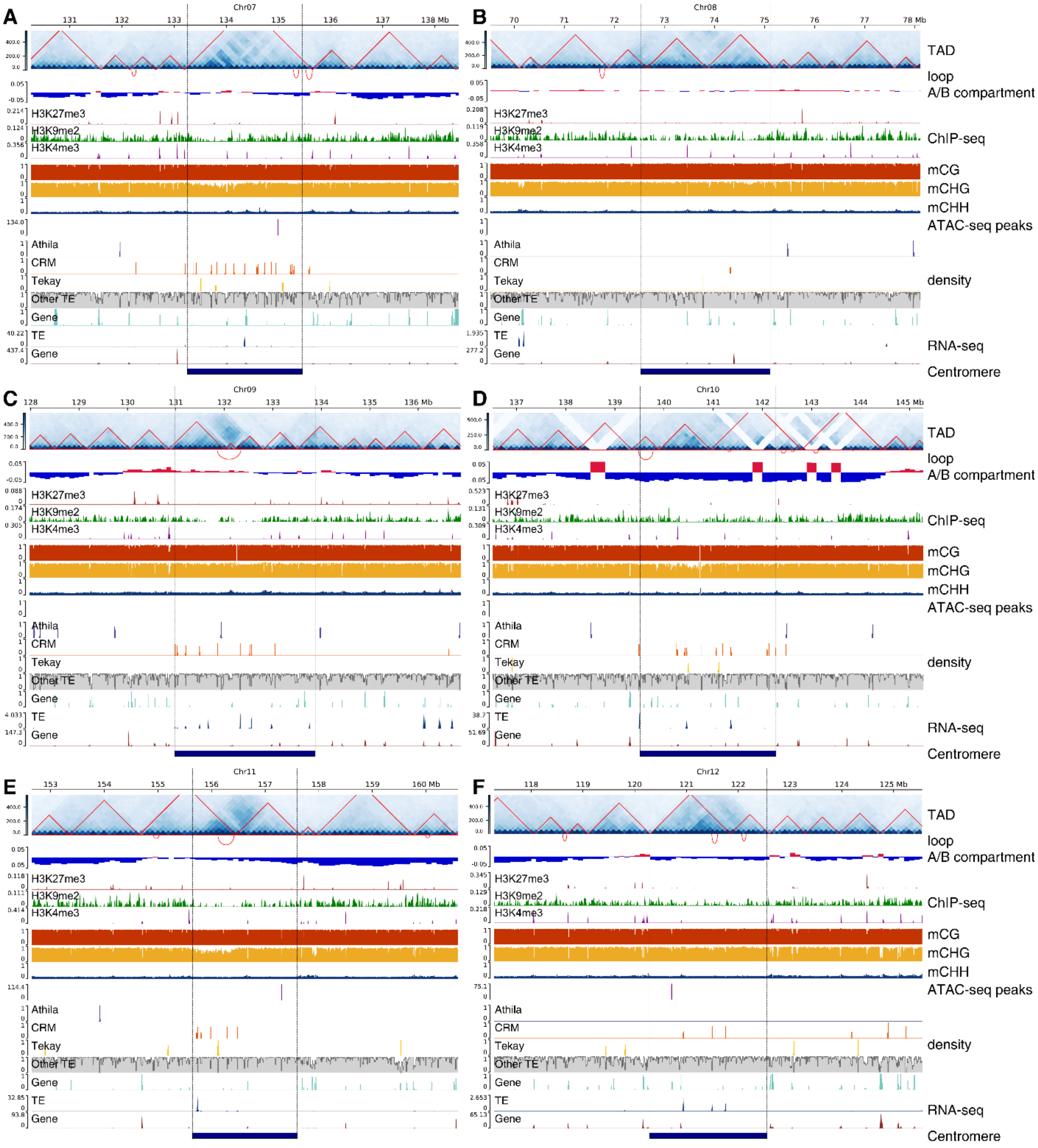

Supplementary Fig. 17. Characteristics of the centromeres on Chr07-Chr12 of *C. annuum*. (**A**-**F**) Characteristics of different centromeres in *C. annuum* assembly. The distributions of TAD and chromatin loops, A/B compartment, ChIP-seq signals (H3K27me3, red; H3K9me2, green; H3K4me3, purple), methylation levels (CG, red; CHG, green; CHH, purple), ATAC-seq signal, TE elements (Athila, bule; CRM, yellow; Tekay, red; others, grey), gene density (green), TE transcript abundances (blue) and gene transcript abundances (red) were plotted from the top to bottom successively. The centromere region was marked by the bottom blue rectangle.

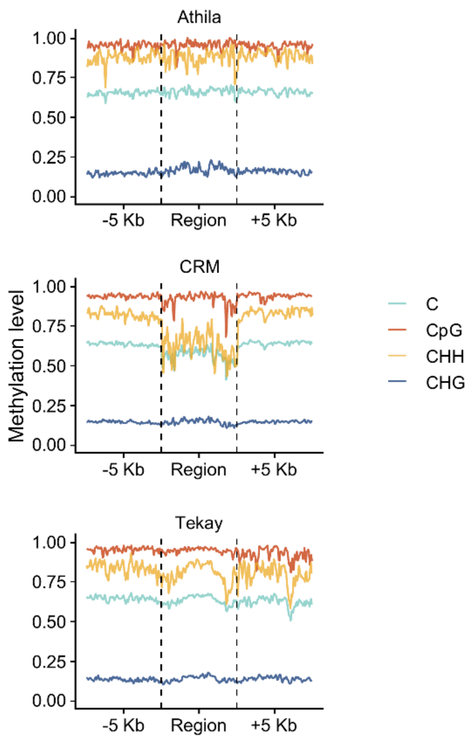

Supplementary Fig. 18. Methylation levels of centromere-located *Athila*, *Tekay* and *CRM* including the upstream and downstream 5 kb regions in *C. annuum*.

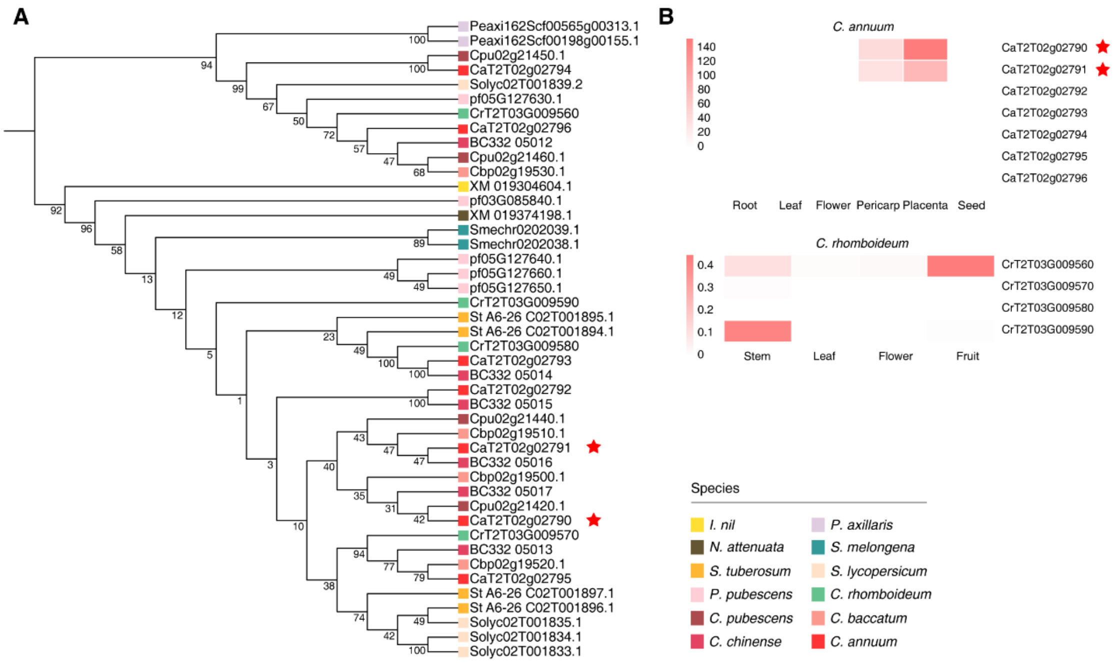

Supplementary Fig. 19. A neighbor-joining phylogenetic tree of capsaicin synthase (CS) genes in representative species of Solanaceae plants. (**A**). Neighbor-joining phylogenetic tree of CS family. The two C. annuum CS genes with placenta-specific high expression are marked with red stars. The support value was placed on the branch with bootstrap (n=1000). (**B**). Gene expression profiles (in normalized TPMs) of different tissues in two species are presented in the heatmap alongside the gene names.

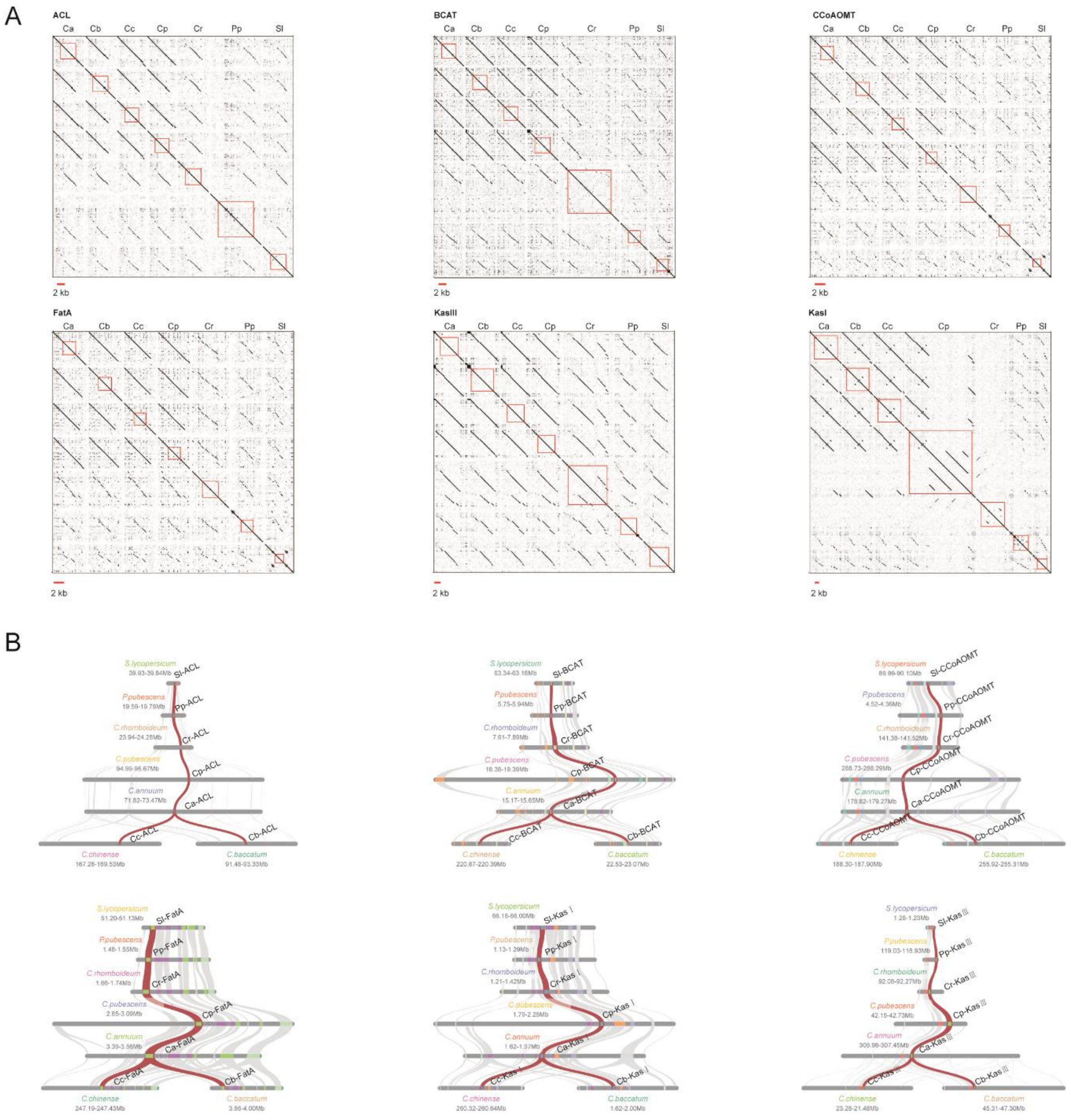

Supplementary Fig. 20. Microsynteny analysis of key CBGs present in a syntenic block conserved among various Solanaceae species. (**A**) Dot plot of ACL, BCAT, CCoAOMT, FatA, KasIII and KasI among various Solanaceae species. Ca, *C. annuum*; Cb, *C. bacatum*; Cc, *C. chinense*; Cr, *C. rhomboideum*; Pp, *Physalis pubescens*; and Sl, *Solanum lycopersicum*. (**B**) Microsynteny relationships of ACL, BCAT, CCoAOMT, FatA, KasIII and KasI among various Solanaceae species. The red lines denote the conserved CBGs among different species.

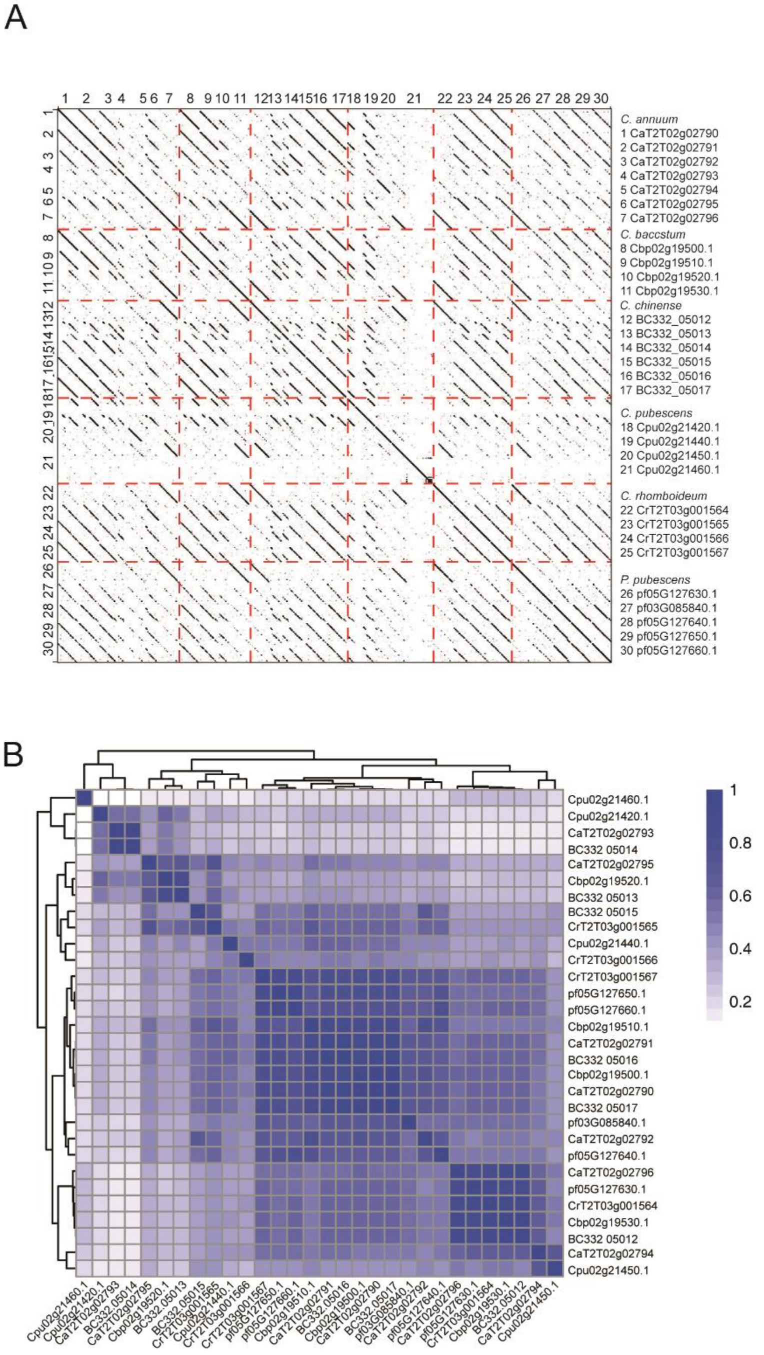

Supplementary Fig. 21. Nucleotide dot plot (A) and sequence identity heatmap (B) of capsaicin synthase and its gene copies from different *Capsicum* species and a close relative *Physalis pubescens*.

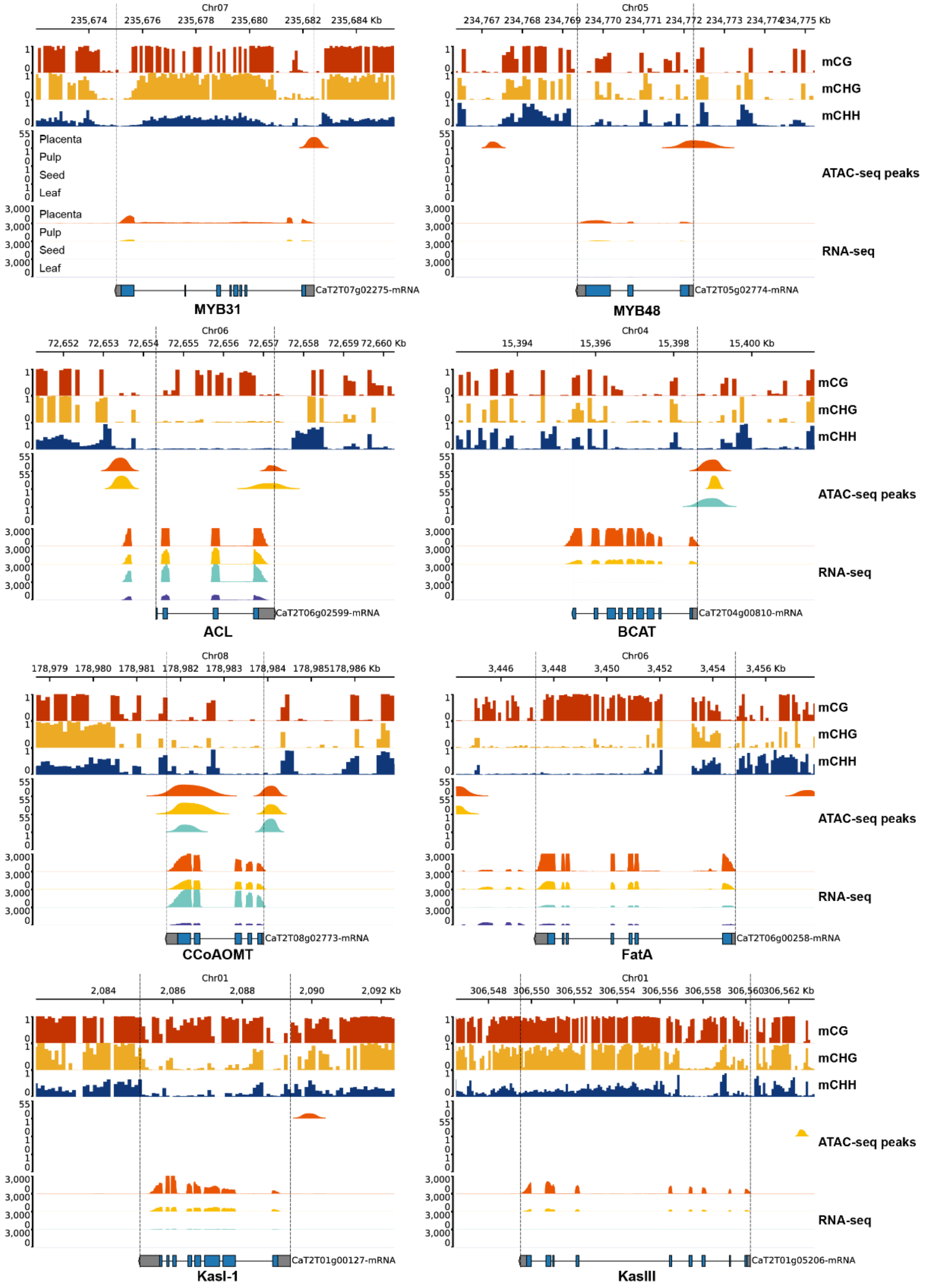

Supplementary Fig. 22. Epigenomic and transcriptomic patterns of two functionally characterized transcription factors (*MYB31*, *MYB48*) and six putative CBGs (*ACL*, *BCAT*, *CCoAOMT*, *FatA*, *KasI-1* and *KasIII*).

Supplementary Table 1. Statistics of HiFi, ONT, NGS and Hi-C sequencing data.

	Total reads	Total bases	Coverage	Avg_len	N50_len
Capsicum annuum					
HiFi	19,053,754	353,996,424,711	114.07	18,578	18,571
HiFi >12kb	19,022,625	353,743,940,045	113.99	18,596	18,575
ONT	8,117,654	261,581,202,255	84.29	32,223	70,093
ONT>20kb	3,529,497	229,079,571,995	73.82	64,904	80,483
NGS	2,005,477,476	300,821,621,400	96.94	150	150
Hi-C	2,321,312,266	348,196,839,900	112.21	150	150
Capsicum rhomboideum					
HiFi	16,336,062	273,450,376,395	159.91	16,739	16,525
ONT	5,124,923	211,994,847,898	123.97	41,365	51,206
NGS	2,829,745,284	424,461,792,600	248.22	150	150
Hi-C	5,409,287,476	809,071,486,706	473.14	150	150

The coverage was calculated using an estimated genome size of 3.10 Gb for *C. annuum* and 1.71 Gb for *C. rhomboideum.*

Supplementary Table 2. Summary statistics from the genome assembly processes.

	HiFi assembly	ONT assembly	Hybrid assembly	rDNA assembly	Final T2T assembly
C. annuum					
Contig number	245	49	226	12	12
Contig size	3,119,947,445	3,097,222,750	3,117,217,487	3,101,167,277	3,103,116,129
Longest contig	338,152,478	280,764,734	338,262,553	338,262,553	338,262,553
Shortest contig	17,870	146,479	17,870	178,376,335	178,437,200
Contig N50	262,400,760	177,848,223	262,559,871	262,559,871	262,573,928
Contig N90	174,125,340	54,150,501	178,376,335	187,569,841	187,643,412
Telomere number	18	22	22	24	24
C. rhomboideum					
Contig number	272	82	111	-	13
Contig size	1,726,076,663	1,740,911,907	1,718,832,306	-	1,708,362,934
Longest contig	172,950,363	144,383,460	172,950,363	-	172,950,363
Shortest contig	12,045	98,391	14,706	-	67,892,047
Contig N50	111,259,455	56,905,120	145,987,823	-	145,987,823
Contig N90	60,239,001	17,927,516	111,259,455	-	111,259,455
Telomere number	17	8	17	-	17

Supplementary Table 3. The closed gaps in HiFi assembly compared to the final T2T assembly.

Chr	Start	End	Length	Gap type
C. annuum				
Chr02	96,789,269	96,767,333	-21,936	
Chr04	251,341,246	250,308,149	-1,033,097	
Chr06	238,689,729	238,693,350	3,621	
Chr06	250,773,616	250,587,875	-185,741	
Chr06	251,506,558	251,319,315	-187,243	
Chr06	251,844,251	251,769,406	-74,845	
Chr06	252,616,557	252,594,115	-22,442	
Chr07	268,635,888	268,649,945	14,057	
Chr08	0	13,406,363	13,406,363	NOR region
Chr11	11,596,486	11,540,566	-55,920	
Chr11	12,216,961	12,163,519	-53,442	
C. rhomboideum				
Chr01	70,164,547	70,146,139	-18,408	
Chr02	61,452,958	61,429,688	-23,270	
Chr02	121,478,287	121,478,485	198	
Chr04	28,183,996	28,150,370	-33,626	
Chr04	127,898,539	127,899,333	794	
Chr09	385,738	389,337	3,599	
Chr09	96,006,130	95,985,645	-20,485	
Chr10	113,117,390	113,118,326	936	
Chr12	224,503	224,484	-19	

Supplementary Table 4. The collinearity at the gene and DNA levels between different assemblies.

	Identity	Ca59	*C.baccatum*	*C.chinense*	CrT2T	*P. pubescens*
Compared with others based on gene synteny						
CaT2T	-	71.55%	49.64%	56.13%	46.38%	43.77%
CrT2T	-	44.24%	32.82%	38.77%	-	38.18%
Compared with others based on DNA level						
CaT2T	No match:	1.89%	18.47%	9.16%	94.50%	97.41%
	< 25%:	0.15%	15.72%	0.93%	5.41%	2.55%
	< 50%:	0.93%	6514%	57.92%	0.09%	0.02%
	< 75%:	7.79%	0.66%	31.95%	0	0.01%
	> 75%:	89.24%	0.01%	0.03%	0	0.01%
CrT2T	No match:	90.03%	91.89%	90.20%	-	94.27%
	< 25%:	9.86%	8.00%	9.68%	-	5.71%
	< 50%:	0.11%	0.11%	0.12%	-	0.02%
	< 75%:	0	0	0	-	0
	> 75%:	0	0	0	-	0

Supplementary Table 5. Statistics for the read coverage against final assemblies.

	NGS coverage	HiFi coverage	ONT coverage
C. annuum			
Chr01	99.96%	100.00%	100.00%
Chr02	99.96%	100.00%	100.00%
Chr03	99.96%	100.00%	100.00%
Chr04	99.96%	100.00%	100.00%
Chr05	99.96%	100.00%	100.00%
Chr06	99.96%	100.00%	100.00%
Chr07	99.96%	100.00%	100.00%
Chr08	99.96%	100.00%	100.00%
Chr09	99.96%	100.00%	100.00%
Chr10	99.94%	100.00%	100.00%
Chr11	99.96%	100.00%	100.00%
Chr12	99.96%	100.00%	100.00%
C. rhomboideum			
chr1	99.96%	100.00%	100.00%
chr2	99.94%	100.00%	100.00%
chr3	99.96%	100.00%	100.00%
chr4	99.93%	100.00%	100.00%
chr5	99.96%	100.00%	100.00%
chr6	99.96%	100.00%	100.00%
chr7	99.96%	100.00%	100.00%
chr8	99.96%	100.00%	100.00%
chr9	99.96%	100.00%	100.00%
chr10	99.94%	100.00%	100.00%
chr11	99.96%	100.00%	100.00%
chr12	99.96%	100.00%	100.00%
chr13	99.96%	100.00%	100.00%

Supplementary Table 6. Summary of closed gaps in CaT2T assembly compared to Ca59 draft genome.

Gap type	Gap number	Longest gap	Shortest gap	Total gap length	Covered genes
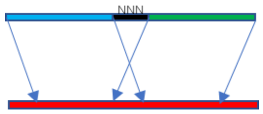 'overlap'	260	553.6 kb	71 bp	7.0 Mb	244
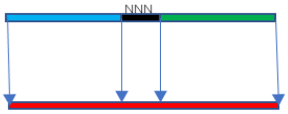 'gap'	175	2.14 Mb	12 bp	21.3 Mb	376
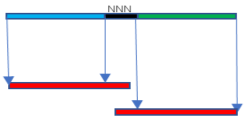 'others'	70	-	-	-	-
Total Gaps	505	2.14 Mb	12 bp	28.3 Mb	614

Gap type was determined by mapping the Ca59 contigs to the CaT2T final assembly.

Supplementary Table 7. Statistics of nuclear mitochondria DNA (NUMTs) and nuclear plastid DNA (NUPTs) in pepper and *Physalis grisea* assemblies. Only the segments longer than 40 kb were counted.

Assembly	NUMTs			NUPTs			Total size
	**Site**	**Size**	**Longest**	**Site**	**Size**	**Longest**	
P. grisea	2	0.14 Mb	94.7 kb	6	0.34 Mb	90.2 kb	0.48 Mb
Ca59	12	0.99 Mb	171.4 kb	16	1.30 Mb	243.7 kb	2.29 Mb
CaT2T	15	1.46 Mb	351.8 kb	18	1.59 Mb	366.3 kb	3.05 Mb
CrT2T	2	0.23 Mb	186.4 kb	6	0.64 Mb	328.4 kb	0.87 Mb

The public genome for *Physalis grisea* was from He et al. 2023.

Supplementary Table 8. Statistics for repetitive elements in centromere, telomere, closed gaps and whole genome.

Percentage (%)	*C. annuum*			
	Whole genome	Centromere	Telomere	Closed gaps
Non-repetitive sequences	20.5	9.63	24.94	30.69
SINEs	0.33	0.06	0.48	0.45
LINEs	2.07	1.30	3.43	1.18
LTR/Copia	4.63	2.16	3.09	3.67
LTR/Gypsy	49.67	70.61	14.89	35.15
DNA transposons	6.78	1.41	6.52	8.15
Unclassified TE	11.74	12.61	14.91	17.79
Small RNA	0.65	0.03	11.37	0.46
Simple repeats	0.62	0.25	15.54	0.49
Others	3.01	1.94	4.83	1.97

Percentage (%)	*C. rhomboideum*		
	Whole genome	Centromere	Telomere
Non-repetitive sequences	25.36	8.99	24.26
SINEs	0.42	0.05	0.29
LINEs	2.37	1.07	2.08
LTR/Copia	4.13	1.07	1.79
LTR/Gypsy	47.34	71.00	8.87
DNA transposons	7.49	1.77	2.31
Unclassified TE	9.39	13.61	54.39
Small RNA	0.23	0.04	0.25
Simple repeats	0.51	0.20	5.70
Others	2.76	2.20	0.06

Supplementary Table 9. Number of intact LTR retrotransposons in whole genome (WG) and centromeres (CEN).

Class	Clade	*C. annuum*		*C. rhomboideum*		*Solanum tuberosum*	
		**WG**	**CEN**	**WG**	**CEN**	**WG**	**CEN**
Copia	Ale	802	3	607	2	733	5
	Alesia	14	0	44	0	17	0
	Angela	3	0	107	0	13	1
	Bianca	90	0	248	0	51	2
	Ikeros	44	0	106	1	16	0
	Ivana	140	0	191	1	144	2
	SIRE	50	1	23	0	48	0
	TAR	264	1	259	1	153	1
	Tork	84	0	213	0	60	0
Gypsy	Athila	2,149	15	806	0	249	17
	CRM	233	62	821	498	36	22
	Galadriel	54	0	17	1	28	0
	Ogre	274	1	134	0	129	1
	Reina	127	2	137	1	76	4
	Tekay	821	16	3,248	19	537	28
Total		5,149	101	6,961	524	2,290	83

The public genome for *Solanum tuberosum* cv. DM8 was from Yang et al. 2022.

Supplementary Table 10. Non-redundant protein sequences used in the phylogenetic tree

	Database	Accession number
C. annuum	This study	-
C. rhomboideum	This study	-
C. pubescens	http://ted.bti.cornell.edu/pepper/	-
C. baccatum	http://ted.bti.cornell.edu/pepper/	-
C. chinense	https://www.ncbi.nlm.nih.gov/	GCA_002271895.2
Nicotiana attenuata	https://www.ncbi.nlm.nih.gov/	GCF_001879085.1
Ipomoea nil	https://www.ncbi.nlm.nih.gov/	GCF_001879475.1
Physalis pubescens	https://ngdc.cncb.ac.cn/gwh/	GWHANUX00000000
Camellia sinensis	https://ngdc.cncb.ac.cn/gwh/	GWHASIV00000000
Solanum tuberosum	http://www.bioinformaticslab.cn/pubs/	DM8.1
Solanum lycopersicum	http://solomics.agis.org.cn/tomato/	SL5.0
Solanum melongena	https://solgenomics.net/	HQ-1315
Petunia axillaris	https://solgenomics.net/	-
Coffea canephora	https://solgenomics.net/	-
Arabidopsis thaliana	https://www.arabidopsis.org/	Araport11
Vitis vinifera	https://phytozome-next.jgi.doe.gov/	Vitis vinifera v2.1
Oryza sativa	https://riceome.hzau.edu.cn/	MH63RS3
