# Supplementary material for: Two telomere-to-telomere gapless genomes reveal insights into Capsicum evolution and capsaicinoid biosynthesis
Source: Nat Commun. 2024 May 20;15:4295. doi: 10.1038/s41467-024-48643-0 (PMC11106260; doi:10.1038/s41467-024-48643-0)
Supplement: Supplementary file 3 — Description of Additional Supplementary Files [file 41467_2024_48643_MOESM3_ESM.pdf]

Description of Additional Supplementary Files

Supplementary Data 1
Candidate enzymes and transcription factors for capsaicinoid biosynthetic pathway in *C. annuum*

Supplementary Data 2
The location of intact CRM retrotransposon in two pepper assemblies

Supplementary Data 3
Summary information of annotated genes in centromere regions and their expression

Supplementary Data 4
Copy numbers of candidate genes involved in capsaicinoid biosynthetic pathway
