# Supplementary material for: Two telomere-to-telomere gapless genomes reveal insights into Capsicum evolution and capsaicinoid biosynthesis
Source: Nat Commun. 2024 May 20;15:4295. doi: 10.1038/s41467-024-48643-0 (PMC11106260; doi:10.1038/s41467-024-48643-0)
Supplement: Supplementary file 8 — Reporting Summary [file 41467_2024_48643_MOESM8_ESM.pdf]

nature portfolio

Corresponding author(s): Li Guo, Hang He

Last updated by author(s): Apr 25, 2024

Reporting Summary

Nature Portfolio wishes to improve the reproducibility of the work that we publish. This form provides structure for consistency and transparency in reporting. For further information on Nature Portfolio policies, see our Editorial Policies and the Editorial Policy Checklist.

Statistics

For all statistical analyses, confirm that the following items are present in the figure legend, table legend, main text, or Methods section.

n/a	Confirmed	
☐	☒	The exact sample size (*n*) for each experimental group/condition, given as a discrete number and unit of measurement
☐	☒	A statement on whether measurements were taken from distinct samples or whether the same sample was measured repeatedly
☐	☒	The statistical test(s) used AND whether they are one- or two-sided<br>*Only common tests should be described solely by name; describe more complex techniques in the Methods section.*
☒	☐	A description of all covariates tested
☒	☐	A description of any assumptions or corrections, such as tests of normality and adjustment for multiple comparisons
☒	☐	A full description of the statistical parameters including central tendency (e.g. means) or other basic estimates (e.g. regression coefficient) AND variation (e.g. standard deviation) or associated estimates of uncertainty (e.g. confidence intervals)
☐	☒	For null hypothesis testing, the test statistic (e.g. *F*, *t*, *r*) with confidence intervals, effect sizes, degrees of freedom and *P* value noted<br>*Give P values as exact values whenever suitable.*
☒	☐	For Bayesian analysis, information on the choice of priors and Markov chain Monte Carlo settings
☒	☐	For hierarchical and complex designs, identification of the appropriate level for tests and full reporting of outcomes
☒	☐	Estimates of effect sizes (e.g. Cohen's *d*, Pearson's *r*), indicating how they were calculated

Our web collection on statistics for biologists contains articles on many of the points above.

Software and code

Policy information about availability of computer code

Data collection	PacBio HiFi reads were processed on PacBio Sequel II system; Nanopore ultra-long reads were collected from GridION X5/PromethION sequencer; Illumina NGS, Hi-C and ChIP-seq reads were collected from Illumina Novoseq 6000 platform; The epigenetics data were downloaded from Liao et al. (4) .
Data analysis	The software used and versions are as follows: Jellyfish (v2.3.0), Genomescope (v2.0), Hifiasm (v0.19.5), NextDenovo (v2.5.0), NextPolish (v1.4.0), Barrnap (v0.9), BWA (v0.7.17), Juicer (v1.5), 3D-DNA (v180419), Juicebox (v1.11.08), BUSCO (v5.4.3), Merqury (v1.3), IGV (v2.12.3), LTR_retriever (v2.9.0), RepeatModeler, RepeatMasker (v4.1.2), TRF (v4.09.1), LTR_Finder (v1.2), LTRharvest (v1.6.2), TEtranscripts, TEsorter (v1.3), minimap2 (v2.24), Winnowmap2 (v2.03), MAKER (v2.31.11), Exonerate (v2.2.0), AUGUSTUS (v3.2.3), HISAT2 (v2.2.1), StringTie (v1.13), BRAKER2 (v2.1.6), Liftoff (v1.6.3), Gffread (v0.12.7), IGV-GSAman (v0.6.76), bowtie2 (v2.5.1), samtools (v1.10), MACS2 (v2.2.7.1), JCVI (v1.1.19), Quickmerge, MCScanX, D-GENIES, OrthoFinder (v2.5.4), TrimAl (v1.4.12), RAxML (v8.2.12), PAML (v4.9), CAFE5, IQ-TREE (v2.0.3), ParaAT (v2.0), Gepard, StainedGlass, Cworld-dekker (v0.0.1), HiC-Pro (v3.1.0), Juicertools (v1.22.01), HiTC (v1.42.0), Bismark (v0.24.0), BWA-MEM (v2.2.1), kallisto (v0.48.0); All codes were deposited at https://github.com/Weikai-47/Pepper_T2T.

For manuscripts utilizing custom algorithms or software that are central to the research but not yet described in published literature, software must be made available to editors and reviewers. We strongly encourage code deposition in a community repository (e.g. GitHub). See the Nature Portfolio guidelines for submitting code & software for further information.

Data

Policy information about availability of data

All manuscripts must include a data availability statement. This statement should provide the following information, where applicable:

- Accession codes, unique identifiers, or web links for publicly available datasets
- A description of any restrictions on data availability
- For clinical datasets or third party data, please ensure that the statement adheres to our policy

The raw sequencing data (PacBio HiFi, ONT, Illumina paired-end, Hi-C and RNA-seq) and genome assembly generated in this study have been deposited in the National Center for Biotechnology Information (NCBI) under accession code PRJNA962192. The genome assembly and annotation files are available at Capsicum Genome Database (http://www.pepperbase.site/node/3) of Peking University Institute of Advanced Agricultural Sciences. Source data are provided with this paper.

Research involving human participants, their data, or biological material

Policy information about studies with human participants or human data. See also policy information about sex, gender (identity/presentation), and sexual orientation and race, ethnicity and racism.

Reporting on sex and gender	n/a
Reporting on race, ethnicity, or other socially relevant groupings	n/a
Population characteristics	n/a
Recruitment	n/a
Ethics oversight	n/a

Note that full information on the approval of the study protocol must also be provided in the manuscript.

Field-specific reporting

Please select the one below that is the best fit for your research. If you are not sure, read the appropriate sections before making your selection.

☒ Life sciences ☐ Behavioural & social sciences ☐ Ecological, evolutionary & environmental sciences

For a reference copy of the document with all sections, see nature.com/documents/nr-reporting-summary-flat.pdf

Life sciences study design

All studies must disclose on these points even when the disclosure is negative.

Sample size	This is not relevant to our study. The plant samples used in this study only included two Capsicum species, a pungent C. annuum 'G1-36576' and its non-pungent wild relative C. rhomboideum 'PI 645680'.
Data exclusions	No data were excluded.
Replication	The samples used for sequencing (PacBio HiFi, ONT, Illumina NGS and Hi-C) were collected from a single plant without replications. The samples used for ChIP-seq sequencing were collected from two biological replications. All attempts at replication were successful.
Randomization	This is not relevant to our study.
Blinding	This is not relevant to our study.

Reporting for specific materials, systems and methods

We require information from authors about some types of materials, experimental systems and methods used in many studies. Here, indicate whether each material, system or method listed is relevant to your study. If you are not sure if a list item applies to your research, read the appropriate section before selecting a response.

Materials & experimental systems

n/a	Involved in the study
☐	☒ Antibodies
☒	☐ Eukaryotic cell lines
☒	☐ Palaeontology and archaeology
☒	☐ Animals and other organisms
☒	☐ Clinical data
☒	☐ Dual use research of concern
☐	☒ Plants

Methods

n/a	Involved in the study
☐	☒ ChIP-seq
☒	☐ Flow cytometry
☒	☐ MRI-based neuroimaging

Antibodies

Antibodies used: The antigen with full peptide sequence corresponding to CaCENH3 was used to produce the anti-CENH3 antibodies in rabbit. The preparation and affinity purification of antisera were conducted by the AtaGenix (Wuhan, China).

Validation: The specificity of the anti-CENH3 antibodies was validated using western blot of total proteins extracted from fresh leaves of C. annuum. The validation was also performed by the AtaGenix (http://www.atagenix.com/).

Plants

Seed stocks: Capsicum annuum double haploid line 'G1-36576' were kindly provided by East-West Seed Group. Capsicum rhomboideum 'PI 645680' was obtained from Fatalii Seeds supplier.

Novel plant genotypes: n/a

Authentication: n/a

ChIP-seq

Data deposition

☒ Confirm that both raw and final processed data have been deposited in a public database such as GEO.

☒ Confirm that you have deposited or provided access to graph files (e.g. BED files) for the called peaks.

Data access links
May remain private before publication.
CENH3 Ilumina ChIP-seq reads from CaT2T have been deposited in the NCBI under the Bioproject (PRJNA962192).
The BED files used for peak calling have been uploaded to GitHub (https://github.com/Weikai-47/Pepper_T2T).

Files in database submission:
DH.CENH3.chip1_R1.fastq.gz
DH.CENH3.chip1_R2.fastq.gz
DH.CENH3.chip2_R1.fastq.gz
DH.CENH3.chip2_R2.fastq.gz
DH.CENH3.input_R1.fastq.gz
DH.CENH3.input_R2.fastq.gz

Genome browser session (e.g. UCSC): No longer applicable.

Methodology

Replicates: Two biological repeats of CENH3 ChIP-seq were conducted;
The input for ChIP-seq was analyzed and sequenced once.

Sequencing depth: The ChIP-seq of CENH3 was sequenced at 3.6x depth for each biological repeat (~67,588,076 reads);
The ChIP-seq of Input was sequenced at 2.2x depth (41,293,908 reads).

Antibodies: The antigen with full peptide sequence corresponding to C. annuum CENH3 was used to produce the C. annuum anti-CENH3 antibodies in rabbit.

Peak calling parameters: The reads were aligned to the genome assembly using bowtie2 with the following parameters: --verysensitive --no-mixed --no-discordant -k 10 --maxins 800. Alignment duplications were marked with picard and were filtered with samtools (view -q 30 -F 2308).

	The peak calling was conducted by MACS2 with parameters: -g 2.5e9 --keep-dup auto --broad --bdg.
Data quality	A total of 80 peaks were above 5-fold enrichment and at FDR 5%.
Software	ChIP-seq mapping and peaks calling were run with the commands, using bowtie2 (v2.5.1), samtools (v1.10), and MACS2 (v2.2.7.1)
